# Use of rituximab in connective tissue disease-associated interstitial lung disease: a narrative review

**DOI:** 10.3389/fmed.2025.1555442

**Published:** 2025-05-14

**Authors:** Chenhao Xu, Zewei Xu, Jin Ren

**Affiliations:** ^1^Department of Respiratory and Critical Care Medicine, The Second Hospital of Jilin University, Changchun, China; ^2^Department of Cardiology, The Second Hospital of Jilin University, Changchun, China

**Keywords:** rituximab, efficacy, connective tissue disease-associated interstitial lung disease, B cell, review

## Abstract

This narrative review examines the therapeutic potential of rituximab, a monoclonal antibody targeting CD20 antigens, for treating connective tissue disease-associated interstitial lung disease. It outlines how rituximab offers a promising therapeutic option, particularly for patients who exhibit limited responses to standard therapies like glucocorticoids and immunosuppressive agents. Rituximab’s mechanism of action, involving B lymphocyte depletion, contributes to attenuated inflammation and may slow pulmonary fibrosis progression. The article synthesizes findings from studies assessing rituximab’s effects on lung function, clinical outcomes, and safety across distinct subtypes of connective tissue disease. It also discusses differential treatment responses based on disease characteristics and pathological subtypes, noting evidence that rituximab may be more effective as an initial treatment in some cases, though further investigation into long-term efficacy remains essential. Despite some associated risks, particularly infections, rituximab generally presents a favorable safety profile compared with conventional immunosuppressive therapies. Future research directions include optimizing dosing protocols, treatment intervals, and patient selection criteria, with emphasis on conducting rigorous, long-term randomized controlled trials to more definitively establish rituximab’s role in managing interstitial lung disease in the context of connective tissue diseases.

## 1 Introduction

Connective tissue disease-related interstitial lung disease (CTD-ILD) encompasses a group of autoimmune disorders, occurring in various types of connective tissue diseases (CTD), such as rheumatoid arthritis (RA), primary Sjögren’s syndrome (pSS), systemic sclerosis (SSc), anti-synthetase syndrome (ASS), and systemic lupus erythematosus (SLE). These diseases exhibit varying levels of pulmonary interstitial inflammation and fibrosis (1). Patients initially may experience symptoms such as dyspnea and cough. As the disease progresses and lung function deteriorates, respiratory failure and even death may ensue. ILD is a heterogeneous group of diseases that differ in etiology, clinical manifestations, radiographic and pathological features, disease progression, and response to treatment (2). However, aberrant immune system activation and sustained inflammatory responses characterize the primary pathological processes (3). Currently, there is no unified standard for treating CTD-ILD. The traditional approach is to treat patients with a combination of glucocorticoids (GC) and immunosuppressive agents. Commonly used immunosuppressive agents include cyclophosphamide (CYC), mycophenolate mofetil (MMF), and azathioprine (AZA). However, some patients may not respond favorably to this treatment or experience significant adverse effects. Rituximab (RTX), as a monoclonal antibody targeting CD20 antigens, can deplete B lymphocytes (B cells) and inhibit aberrant immune system activation. RTX has been widely used in the treatment of RA, SSc, and other rheumatic diseases (4). Recently, it has also been explored for treating CTD-ILD, with several large-scale clinical trials completed (5–25). This narrative review aims to comprehensively assess the existing literature on RTX’s efficacy and safety in treating CTD-ILD and briefly describe the role of B cells, as target cells of rituximab, in the development of CTD-ILD. It will focus on three key areas: the impact of RTX on lung function in different CTD types, its safety profile, and the potential factors that may influence the efficacy of RTX. Finally, it will offer insights into RTX’s prospects in treating CTD-ILD.

## 2 Methods

We conducted a comprehensive literature search of the PubMed (MEDLINE) database for terms “Rituximab,” “interstitial lung disease,” “interstitial pneumonia,” “lung fibrosis,” “rheumatoid arthritis,” “systemic sclerosis,” “primary Sjögren syndrome,” “systemic Lupus erythematosus,” “anti-synthetase syndrome,” and “connective tissue diseases,” using the Boolean operator of AND or OR. The majority of the included articles were published in peer-reviewed journals within the last 5 years, and the types of articles included clinical trials, observational studies, and case reports. At the same time, all citations and cited articles of the article were fully searched and analyzed (23).

Key articles of RTX in CTD-ILD are summarized in Table 1.

**Table 1:** Key articles of RTX in CTD-ILD.

References	Study type	Sample size	RTX dosage	Follow-up duration	Lung function stability or improvement	Serious adverse events
RA-ILD						
Narvaez et al. (5)	Longitudinal retrospective observational study	31	1 g, Day 1 and Day 15	12 months	Yes	Yes
Matson et al. (6)	Multisite retrospective cohort study	43 RTX / 92 AZA / 77 MMF	1 g, Day 1 and Day 15	12 months	Yes	No
Yusof et al. (7)	Single-center retrospective observational study	56	1 g, Day 1 and Day 14	12 months	Yes	Yes
Mankikian et al. (8)	Multicenter, double-blind, randomized, placebo-controlled, phase III trial	3 (included in other CTD-ILD patients)	1 g, Day 1 and Day 15	6 months	Yes	No
pSS-ILD						
Kesireddy et al. (9)	Case report	-	1 g, Day 1 and Day 14	1 months	Symptoms improved, no need for oxygen	-
Chen et al. (10)	Retrospective cohort study	10	1 g, Day 1 and Day 14	6 months	Yes	No
Manikuppam et al. (11)	Retrospective cohort study	2	1 g, Day 1 and Day 14	6 months / 12 months	Stable at 6 months / Worsened at 12 months	Yes
SSc-ILD						
Sircar et al. (12)	Open-label, randomized, controlled trial	30 RTX / 30 CYC	1 g, Day 0 and Day 15	6 months	Yes	Yes
Maher et al. (13)	Double-blind, double-dummy, randomised, controlled, phase IIb trial	37 (included in other CTD-ILD patients)	1 g, Day 1 and Day 14	24 weeks / 48 weeks	Yes	No
Narvaez et al. (14)	Longitudinal retrospective observational study	24	1 g, Day 1 and Day 15	1 year / 2 years	Yes	Yes
Lepri et al. (15)	Retrospective multicenter cohort study	23 (included in other CTD-ILD patients)	-	1 year / 2 years	Yes	Yes
Ebata et al. (16)	Open-label extension of a double-blind, investigators-initiated, randomized, placebo-controlled trial	28 RTX / 26 non-RTX	375 mg/m^2^, once per week for 4 consecutive weeks	24 weeks	Yes	No
ASS-ILD						
Sem et al. (17)	Retrospective case series	11	1 g, Day 0 and Day 14 / 700 mg, Day 0 and Day 14 / 375 mg/m^2^, once per week for 4 consecutive weeks	3–6 months	Yes	Yes
Doyle et al. (18)	Multicenter retrospective cohort study	25	-	1 year / 2 years / 3 years	Yes	Yes
Allenbach et al. (19)	Open-label, single-arm, phase II clinical	10	1 g, Day 0, Day 15, and Month 6	6 months / 12 months	Yes	No
SLE-ILD						
Lim et al. (20)	Case report	-	375 mg/m^2^, once per week for 4 consecutive weeks	3 months	Yes	-
Keir et al. (21)	Retrospective cohort study	1 (included in other CTD-ILD patients)	1 g, Day 0 and Day 14	6–12 months	Yes	Yes
Robles-Perez et al. (22)	Retrospective observational study	4 (included in other CTD-ILD patients)	1 g, Day 0 and Day 14 every 6 months	1 year / 2 years	Yes	No
RTX+NTB in CTD-ILD						
Boutel et al. (23)	Retrospective observational study	1 RTX + NTB (included in other patients treated with immunosuppressives and NTB)	-	10 months	Yes	Yes (gastrointestinal events)
Mısırcı et al. (24)	Multicenter retrospective study	18 RTX + NTB (included in other patients treated with immunosuppressives and NTB)	-	6 months / 12 months / 18 months	Yes	No
Ushio et al. (25)	Retrospective cohort study	6 RTX + NTB (included in other patients treated with immunosuppressives and NTB) / 15 NTB (non-IS)	-	8 months	Yes	Yes (gastrointestinal events)

CTD-ILD, Connective Tissue Disease-Associated Interstitial Lung Disease; RA-ILD, Rheumatoid Arthritis-Associated Interstitial Lung Disease; pSS-ILD, Primary Sjögren’s Syndrome-Associated Interstitial Lung Disease; SSc-ILD, Systemic Sclerosis-Associated Interstitial Lung Disease; ASS-ILD, Antisynthetase syndrome-associated interstitial lung disease; SLE-ILD, Systemic Lupus Erythematosus-Associated Interstitial Lung Disease; RTX, Rituximab; AZA, Azathioprine; MMF, Mycophenolate mofetil; CYC, Cyclophosphamide; NTB, Nintedanib.

## 3 The role of B cells in CTD-ILD

B cells play a pivotal role in the pathogenesis of CTD-ILD. Their involvement is mediated through four primary mechanisms: antibody production, antigen presentation, cytokine secretion, and collaboration with other immune cells. In SSc and SLE-related ILD, patients often present with elevated self-antibody levels, including anticentromere and antinuclear antibodies, as well as other antibodies. These antibodies bind to antigens, forming immune complexes that accumulate in the lungs, triggering complement activation and inflammatory responses, ultimately damaging lung tissue. Research has demonstrated that in CTD, the presence of high levels of self-antibodies in ILD patients’ lung lavage fluid indicates that B cells play a pivotal role in the development of CTD (26–28). B cells, acting as antigen-presenting cells, interact with T cells, thereby stimulating the activation of CD4 + regulatory T cells(Th1,Th17), which secrete pro-inflammatory cytokines, further exacerbating lung damage. Additionally, they can activate fibroblast cells, contributing to fibrosis progression (29). B cells also secrete a range of pro-inflammatory and anti-inflammatory cytokines, including IL-10, IL-35, TGF-*β*, IL-6, INF-*γ*, GM-CSF, etc. Of particular significance in the context of pulmonary fibrosis are TNF-*α*, IL-6, and TGF-β, which have been identified as critical mediators in the initiation and maintenance of lung fibrosis. They induce the activation of myofibroblast and excessive deposition of extracellular matrix, ultimately leading to irreversible pulmonary fibrosis (30, 31).

B cell depletion therapy targets specific receptors on the surface of B cells, particularly CD20, thereby removing or inhibiting abnormally active B cells, reducing the production of autoantibodies, and attenuating the immune response, ultimately effectively inhibiting fibrosis. Rituximab is one of the commonly used B cell depletion drugs (32). SSc has been the focus of numerous studies on the role of immunosuppressive agents and the use of RTX in treating ILD. Research findings indicate that B cells with high affinity for topoisomerase I (topo I) produce pro-inflammatory factors, including IL-6 and IL-23, and induce pro-inflammatory Th17 polarization. This, in turn, promotes the development of lung fibrosis. The level of B cells with high affinity for topo I in SSc is closely correlated with the severity of the disease. RTX slows progression of pulmonary fibrosis by depleting these B cells and restoring immune system balance. In addition, in a mouse model of bleomycin-induced SSc, B cells promote the differentiation of macrophages towards a pro-fibrotic M2-type. This process can be inhibited through B cell depletion, thereby attenuating lung fibrosis. In addition to RTX, other therapies targeting B cells, such as ibrutinib and CAR-T cell therapy, have demonstrated potential in treating CTD-ILD. Ibrutinib, a bruton tyrosine kinase (BTK) inhibitor, interferes with B cells signaling, inhibits B cells activation, and reduces fibrosis. CAR-T cell therapy reduces fibrosis by targeting CD19 or B cell receptorto (BCR) remove overactive B cells directly (33). B cell depletion provides additional therapeutic options for the treatment of CTD-ILD.

## 4 Rituximab in the treatment of CTD-ILD

### 4.1 RA-ILD

ILD is a common cause of mortality in RA, accounting for 10–30% of cases (34). It is the second most prevalent cause of death in RA patients after cardiovascular factors. The one-year and five-year mortality rates for RA-ILD have been reported to be 13.9% and 39% (35), respectively. UIP and NSIP are the most prevalent pathological subtypes of RA-ILD, and the disease often manifests insidiously. It can be observed that the disease can manifest at any stage of the RA disease process or concurrently with RA. Currently, there is no unified standard of care. The most commonly used treatments are glucocorticoids and (or) immunosuppressive agents (such as MMF, AZA, and CYC). Prior studies indicated that methotrexate (MTX) and leflunomide (LEF) should be avoided due to their pulmonary toxicity (36). However, recent mainstream studies have demonstrated that methotrexate use does not increase the risk of RA-ILD development and progression. Instead, methotrexate use may offer a protective effect against RA-ILD development (37, 38).

The findings of Javier Narváez et al. (5) indicate that RTX is effective in treating refractory and progressive RA-ILD. There were significant improvements in pulmonary function test (PFT) compared to baseline, with an absolute change of +8.06% in FVC% (95% CI: −10.9 to −5.2; *p* < 0.001) and +12.7% in DLCO% (95% CI: −16.3 to −9.1; *p* < 0.001). Additionally, It was found that there was no significant difference in PFT improvement between the UIP and non-UIP groups after treatment. At the end of the follow-up period, 32% of patients experienced adverse reactions, primarily involving respiratory and urinary tract infections and low serum levels of IgG (5). RTX not only proved effective in treating RA-ILD as a salvage therapy (5) after initial treatment failure but also demonstrated efficacy when used as the initial treatment (6). Matson et al. (6) reported using rituximab for RA-ILD in a multicenter retrospective cohort study. The impact of initial treatment on PFT was evaluated in a cohort of 212 patients who received either AZA, MMF, or RTX. At 12 months post-treatment, all patients exhibited significant improvements in FVC% (3.90%; *p* < 0.001; 95% CI, 1.95–5.84) and DLCO% (4.53%; *p* < 0.001; 95% CI, 2.12–6. 94), with RTX treatment resulting in a more remarkable improvement in DLCO% than AZA and MMF treatment. The incidence of adverse events leading to treatment cessation was also lower in the RTX group than in the AZA and MMF groups (1.3% vs. 13% vs. 3.9%). Furthermore, subgroup analysis revealed that the UIP pattern did not influence the efficacy of RTX treatment in the RTX group or the other two groups (6). Md Yuzaiful et al. (7) noted that patients with RA-ILD who received RTX treatment exhibited stabilization or improvement of PFT. However, patients with UIP patterns demonstrated inferior treatment responses, with more progression of ILD and a poorer prognosis. Conversely, patients with NSIP patterns exhibited superior treatment responses and outcomes. Among the 56 patients who received RTX treatment, nine patients exhibited severe progression of ILD, which may be attributed to their lower DLCO% (median 41%) prior to treatment or UIP patterns, and 12 patients exhibited severe infection, with 5 of these patients concurrently receiving glucocorticoid therapy. Through survival analysis, it was determined that UIP, a history of prior ILD progression, and pretreatment DLCO% levels below 46% could effectively predict the progression of ILD post-treatment (*p* = 0.02, *p* = 0.001, *p* = 0.001). Given these findings, the researchers concluded that RTX remained a promising option. The safety profile is satisfactory (7). Currently, no RCT has been conducted specifically to evaluate the efficacy and safety of RTX in treating RA-ILD. Mankikian et al. (8) published a Phase III clinical trial that included three cases of RA-ILD, and all three cases were diagnosed with NSIP. The trial’s objective was to assess the efficacy and safety of RTX in combination with MMF. The results demonstrated that patients with RA-ILD who received combined treatment exhibited better preservation of lung function and improved survival rates compared to those who received MMF alone. There were no severe adverse reactions observed (8).

The findings above suggest that whether used as initial therapy or as a salvage treatment following the failure of the initial therapy, RTX has a positive effect on PFT in RA-ILD patients (5, 6). The incidence of adverse reactions during treatment varies between the studies, but the results still demonstrate a favorable safety profile (5–8). However, the specific pathological subtype may influence the response to RTX and its safety profile (5–7). The studies by Scott M. Matson, MD, and Javier Narváez, among others, suggest that there was no difference in response to treatment between UIP and non-UIP modes. Md Yusof and colleagues observed that the prognosis of the UIP pattern is worse after treatment, and they also point out that it may be related to the fact that UIP itself is a risk factor for ILD progression. Furthermore, combining RTX with traditional immunosuppressive agents demonstrated superior efficacy compared to monotherapy with immunosuppressive agents alone. Future studies should investigate the differential treatment responses to RTX in different RA-ILD subtypes to address the remaining controversies and identify the optimal timing for RTX use.

### 4.2 pSS-ILD

pSS-ILD has a latent onset and a slow progression. Its prevalence in pSS populations is 9–20% (39). The prognosis is unfavorable, with a higher mortality rate and a 10-year survival rate of 81.7%. Of these patients, 67% die from respiratory failure (40). NSIP is the most common pathological subtypes, others are UIP, OP, and LIP. The traditional treatment drugs are glucocorticoids and (or) immunosuppressive agents (e.g., AZA, CYC, and MMF).

Kesireddy, Nithin MD et al. reported a case of pSS-ILD treated with RTX. The patient in question displayed HRCT characteristics indicative of OP mode. Following the failure of GC + MMF as a first-line treatment, GC + RTX was initiated. After 2 weeks, mechanical ventilation was discontinued, and 1 month later, the patient exhibited a notable improvement in symptoms, obviating the need for oxygen therapy. This case report suggests that RTX may serve as a viable salvage therapy for refractory pSS-ILD (9). A retrospective cohort study by Chen et al. on the efficacy of RTX in 10 pSS-ILD patients revealed that 6 months after treatment, DLCO, DLCO/alveolar volume, and VAS scores showed significant improvement compared to baseline. However, FVC% remained unchanged. Although HRCT scores declined compared to the pretreatment period, the change was not statistically significant. During the follow-up period, only one subject in the experimental group was hospitalized due to pneumonia, and no serious adverse events were reported. The results of this study showed that RTX could safely and effectively improve the clinical symptoms and PFT of pSS-ILD, and stabilize the HRCT score (10). The study above demonstrated that RTX treatment for pSS-ILD patients exhibited superior short-term efficacy (9, 10). However, Manikuppam et al. (11) mentioned that in the context of MMF therapy, patients who are added RTX due to progression of pSS-ILD reached a stable condition at 6 months of follow-up, but at the one-year mark, there was an exacerbation of ILD, and the clinical symptoms remained stable again after increasing the dose of MMF. Additionally, the study identified patients exhibiting an NSIP pattern often own a better treatment response, only one patient succumbed to bacteremia, and no significant adverse reactions were observed in the remaining patients. This outcome may be attributed to administering co-trimoxazole and vaccines prior to treatment (11). Currently, NSIP and OP demonstrate more favorable treatment outcomes. However, further extended follow-up periods are necessary to monitor RTX’s long-term efficacy and assess the optimal combination of immunosuppressive agents, such as MMF, for more effective treatment (9–11).

The studies above suggest that RTX may be a viable option for treating refractory pSS-ILD (9). Using RTX in monotherapy or combination with MMF has been shown to have favorable outcomes regarding clinical symptoms, PFTs, and HRCT scores (11). However, it is essential to monitor the long-term efficacy of this treatment. RTX has been observed to have an improved safety profile, which may be attributed to the preventive measures taken prior to treatment (11). Additionally, the different pathological subtypes of interstitial lung disease may respond differently to RTX (9, 11). The efficacy of RTX in UIP patterns remains uncertain and requires further evidence.

### 4.3 SSc-ILD

SSc-ILD represents the most common cause of mortality in SSc patients. NSIP is the most prevalent pathological subtype, followed by OP and UIP. Approximately 50–80% of patients present with concomitant ILD (41). Patients with diffuse cutaneous systemic sclerosis (dcSSc) exhibit a higher risk of developing ILD, which can be identified at an early stage of the disease and subsequently progresses to pulmonary fibrosis, ultimately leading to respiratory failure. Because the use of GC has been associated with the renal crisis, immunosuppressive agents are often used in current practice (42).

In an open-label randomized controlled trial, Geetabali Sircar and colleagues compared the efficacy and safety of intravenous CYC and RTX in the early treatment of diffuse cutaneous systemic sclerosis-related interstitial lung disease. The trial included 60 patients randomly assigned to receive cyclophosphamide (500 mg/m^2^, every 4 weeks) and rituximab (1,000 mg, D0 and D15). Six months later, a more pronounced improvement in FVC % and modified Rodnan skin score (mRSS) was noted in the RTX group compared to the CYC group (RTX group FVC % increased from 61.3% to 67.52%, while the CYC group FVC % decreased from 59.25% to 58.06%, *p* = 0.03. RTX group mRSS scores decreased from 21.77 to 12.10, while the CYC group decreased from 23.83 to 18.33, *p* = 0.01). Additionally, the incidence of severe adverse reactions was significantly lower in the RTX group (30%) compared to the CYC group (70%). This findings suggest that RTX may be the preferred option over CYC in treating early diffuse cutaneous systemic sclerosis-related interstitial lung disease, offering a safe and effective treatment protocol (12). In a recent double-blind, double-placebo, randomized controlled, phase IIb clinical trial published by Toby M. Maher et al., 37 patients with severe or rapidly progressive SSc-ILD were included and randomized to receive CYC (600 mg/m^2^, every 4 weeks) and RTX (1,000 mg, D1 and D14). At the 24 week follow-up, the FVC increased significantly in the CYC and RTX groups compared to the baseline. However, the difference between the two groups was not statistically significant (*p* = 0.49). Gastrointestinal and respiratory disorders were the most common adverse events during treatment in both groups. However, adverse events, including serious ones, occurred more frequently in the CYC group than in the RTX group. Notably, during the treatment period, the RTX group received a lower daily GC dosage (37.6 mg vs. 42.9 mg), suggesting that the probability of renal crisis due to elevated GC use was diminished. While RTX did not demonstrate a statistically significant difference compared to CYC in terms of the primary endpoint of change in FVC, it exhibited notable benefits, including a reduction in adverse events, and a decrease in GC use. Consequently, RTX may be regarded as a potential alternative treatment option for the management of severe or rapidly progressive CTD-ILD (13). Similarly, a favorable outcome was also reported by Javier Narváez and et al. (14) whereby the addition of rituximab to a regimen of MMF was found to be efficacious in patients with progressive SSc-ILD who had previously responded inadequately to MMF monotherapy. In these patients, there was a significant improvement in FVC% and DLCO% at the 1-year and 2-year follow-ups. Subgroup analysis revealed that the improvement was limited to patients with non-UIP lung disease patterns, while no statistically significant difference was observed in UIP patients. The incidence of this adverse event was 37.5%, with most cases presenting as infection or low IgG levels. However, only 12.5% of patients discontinued treatment due to severe infection (14). In contrast, in a retrospective study, Lepri et al. (15) reported on 23 SSc-ILD patients treated with rituximab. One-year follow-up revealed an improvement in FVC%, though this was not statistically significant. At the two-year mark, there was a decline in FVC% (15). In the DESIRES study, a multicenter RCT that included 54 patients with mRSS≥10 and expected survival of ≥6 months, randomization was performed to assign them to either receive 375 mg/m^2^ of rituximab or a placebo. The treatment was administered once a week for 4 weeks, and the patients were followed up for 24 weeks. The results demonstrated that the RTX group exhibited a significantly lower mRSS score than the placebo group (mean difference: −8.44). The mean change in FVC% from baseline to 24 weeks was 0.68 (95% CI: −11.0 to −0.36) in the RTX group and −5.88 (95% CI: −8.76 to −3.00) in the placebo group, with a statistically significant difference (*p* < 0.0001). In the RTX group, FVC% increased from week 12 to week 24, while in the placebo group, it continued to decline, with a statistically significant difference (*p* = 0.044). This effect was more pronounced in patients with diffuse cutaneous systemic sclerosis-related interstitial lung disease than those with limited cutaneous systemic sclerosis-related interstitial lung disease. The two groups exhibited similar adverse effects during and after treatment, primarily mild infection, with minimal severe adverse reactions. This findings suggest that rituximab is highly effective in improving skin sclerosis in SSc patients and can potentially stabilize lung function in SSc-ILD patients. This suggests that rituximab may be a promising therapeutic option for treating SSc-ILD (16).

The studies above indicate that RTX positively impacts the progression of early-stage or progressive SSc-ILD (12, 14). The beneficial effects on skin symptoms and lung function were notable, particularly in non-UIP patterns and diffuse cutaneous systemic sclerosis-associated interstitial lung disease (16). The findings of Lepri et al. (15) indicated that RTX did not significantly enhance lung function, there was a deterioration in lung function during the second year of treatment. Therefore, further investigation is required to ascertain the efficacy and safety of RTX in treating different subtypes of SSc-ILD.

### 4.4 ASS-ILD

ASS is a subtype of idiopathic inflammatory myopathy characterized by the production of anti-synthetase antibodies (such as anti-Jo-1, PL-7, PL-12, EJ, and OJ). It presents clinically with myositis, polyarthritis, Raynaud’s phenomenon, scleroderma, and ILD syndrome. Compared to Polymyositis/ Dermatomyositis, ASS is more frequently associated with ILD. NSIP is the most common pathological subtype (43). The current standard of care is a combination of glucocorticoids and (or) immunosuppressive agents.

The results of Marthe Sem’s research indicate that RTX improved or stabilized PFT in seven of 11 refractory, severe ASS-ILD patients over a three-to-six-month period. Additionally, five of the patients showed a reduction in ground-glass opacities on HRCT scans. During the follow-up period, one patient died in pneumocystis jirovecii pneumonia (PJP), else did not experience severe adverse reactions. This study demonstrated that RTX could be an effective and safe alternative treatment for severe progressive ASS-ILD in the short term (17). In a multicenter retrospective study, Tracy J. Doyle et al. reported that RTX was a salvage treatment for 21 refractory ASS-ILD cases and an initial treatment for 4 ASS-ILD cases. In the first year of follow-up, most patients showed improved DLCO%. The percentage of FVC% and DLCO% remained stable or improved, with a significant increase in the percentage of FVC observed in the second and third years of follow-up. This findings indicate that RTX can demonstrate favorable long-term efficacy when used as a salvage or initial treatment for ASS-ILD. Additionally, patients who received continuous and regular RTX treatment (one cycle every six months) demonstrated superior improvements in PFT and HRCT compared to those who received a single cycle of treatment or no regular medication. This suggests that the duration of treatment and the interval between cycles influence the efficacy of RTX. The main complications observed during RTX treatment were infections, including pneumonia, influenza, urinary tract infections, and cellulitis (18). In a phase II clinical trial investigating the efficacy of RTX in 10 cases of refractory ASS-ILD, five patients exhibited improved PFTs (50%, 95% CI: 1–95%) after receiving 1,000 mg of RTX(D0, D15, M6). Four patients exhibited stability, while one exhibited deterioration. The researcher indicated that only two patients demonstrated improvement in DLCO, suggesting that the observed improvement in PFT may be attributed to muscle strength rather than ILD. No adverse events were observed during treatment (19).

These findings suggest that RTX may be an effective intervention for both salvage and initial treatment of ASS-ILD, demonstrating improved short-term and long-term efficacy while maintaining safety. Apart from infection, no other serious adverse events were observed (17, 18). However, Yves and colleagues have suggested that muscle strength gains may influence the improvement in PFT results following RTX treatment (19). Furthermore, Tracy J. Doyle and colleagues have demonstrated that patients receiving different treatment regimens and varying intervals between doses exhibit varying treatment outcomes (18). Currently, the majority of research findings are derived from retrospective studies. To investigate the efficacy and safety of RTX further, as well as the optimal dosage and interval, prospective, randomized controlled trials are needed.

### 4.5 SLE-ILD

It is relatively uncommon for SLE to be combined with ILD, with an incidence rate of 1–15% (44). The most prevalent pathological subtype is NSIP, with other notable examples including OP, UIP, LIP, and diffuse alveolar damage. Currently, there is no established standard treatment plan, with the current approach based mainly on expert opinion and often involving the use of glucocorticoids and (or) immunosuppressive agents (e.g., CYC and MMF) as a first-line induction and maintenance therapy.

In 2006, Lim et al. (20) published the first case report of a patient with refractory SLE-ILD treated with RTX. After switching from immunosuppressive therapy to RTX, the patient improved FVC%, DLCO%, and SLAM scores. The report highlights the potential of rituximab in treating refractory SLE-ILD when conventional therapies are ineffective (20). Similarly, Keir et al. (21) reached a comparable conclusion in a retrospective study, whereby all patients exhibited significant pulmonary function improvements following rituximab treatment. However, a subset of patients did develop serious complications. For patients with SLE-ILD who have failed conventional treatments, RTX may offer a viable intervention. Nevertheless, further prospective studies are necessary to assess the efficacy and safety of the drug. Robles-Perez et al. (22) conducted a similar involving 18 end-stage CTD-ILD patients awaiting transplantation including four cases of SLE-ILD. RTX therapy was administered. Following treatment, there was a significant improvement in lung function, with a further notable improvement in DLCO% at the two-year mark. Adverse effects were primarily respiratory and urinary tract infections. There were no deaths during the observation period, and the need for lung transplantation was delayed. This findings suggest that RTX can be used as a pre-transplant rescue therapy and positively impacts lung function over 2 years. The positive effects observed in this study are encouraging—however, the age limit of 65 years and the small sample size warrant further investigation (22).

SLE-ILD patients are often included in more extensive CTD-ILD studies, which reduces the accuracy of the results. Nevertheless, the combination of case reports and this study’s results suggest that RTX may be beneficial in treating SLE-ILD as a salvage therapy (20–22). However, further research is needed to explore the optimal timing and dosage of RTX.

### 4.6 Rituximab in combination with antifibrotics

In current clinical practice, traditional immunosuppressive agents are still significant in managing CTD-ILD. Several studies have demonstrated that RTX therapy exhibits superior efficacy and safety compared to traditional immunosuppressives. RTX has shown promising therapeutic potential as both a first-line and a salvage treatment. Despite CTD-ILD and IPF differ in clinical features, natural course, treatment, and prognosis, they overlap in pathogenic mechanisms. The disease courses of CTD-ILD can vary, and when patients present with progressive exacerbation of pulmonary fibrosis and deterioration of lung function, they can be defined as having progressive pulmonary fibrosis (PPF) once they meet the diagnostic criteria (45). Nintedanib (NTB), an oral tyrosine kinase inhibitor, exerts its effects by targeting and inhibiting the receptors for platelet-derived growth factor (PDGF), fibroblast growth factor (FGF), and vascular endothelial growth factor (VEGF) (46). This effectively inhibits the proliferation and activation of fibroblasts associated with pulmonary fibrosis, thereby reducing the progression of fibrosis. In two Phase III clinical trials (INPULSIS-1 and INPULSIS-2), it has been demonstrated that NTB treatment significantly slows the decline of FVC in patients with IPF (47). Furthermore, a Phase III clinical trial (INBUILD) has demonstrated the positive efficacy of NTB in PPF (48). Presently, NTB has been approved for the treatment of IPF and PPF. Combining immunosuppressives with NTB is a significant treatment modality in contemporary clinical practice. The most recent CTD-ILD management opinion published by the BSR and EULAR indicates that MMF, in combination with NTB, can be utilized as a treatment for SSc-ILD (49, 50).

Boutel et al. (23) conducted a single-center descriptive study based on real-world data, enrolling 21 patients with CTD-ILD treated with NTB. The majority of these patients were receiving concurrent immunosuppressive therapy, and one patient was treated with RTX in combination with NTB. The 10-month follow-up endpoints revealed lung function remained stable before and after treatment, with a slight numerical improvement (the mean change in FVC was +0.9%, the mean change in DLCO was +3.4%, and the mean change in FEV1 was +3.4%). No serious adverse events were observed. Further statistical analysis showed that patients treated with NTB in combination with MMF had a more significant improvement in DLCO% than those treated with NTB alone (*p* = 0.03), and although the subgroup of RTX combined with NTB was not analyzed, the positive results could indicate that immunosuppressives combined with NTB is effective in slowing down the progression of fibrosis in CTD-ILD (23). Similar conclusions were obtained by Mısırcı et al. (24) in a multicenter, larger sample size, and longer follow-up study in which all patients were treated with immunosuppressives in combination with NTB, of which 27.3% treated with RTX. The FVC and DLCO values remained stable compared to the baseline after 6, 12, and 18 months of treatment, and the study further found that the combination of immunosuppressives and NTB is effective and safe in the treatment of both SSc-ILD patients and other CTD-ILD patients. In another retrospective study, Yusuke Ushio et al. classified the subjects into the immunosuppressives + NTB treatment group and the NTB alone treatment group. The most commonly utilized immunosuppressives in this study was RTX. Following 8 months of treatment, the immunosuppressives + NTB treatment group exhibited a significant improvement in the change in FVC and the rate of change in monthly FVC compared to the NTB alone treatment group (+12.1% vs. −1.1%, +1.71%/month vs. +0.34%/month, respectively). And the immunosuppressives + NTB treatment group experienced a serious adverse event of gastrointestinal perforation during follow-up. This adverse event may be related to the long-term use of GC prior to the patients receiving treatment or the inhibitory effect of NTB on platelet-derived growth factor receptor (VEGFR). Since RTX is the most widely used in immunosuppressives in this study, it indicates that RTX combined with NTB for CTD-ILD treatment of the PPF phenotype demonstrates some potential (25).

The potential of immunosuppressives in combination with NTB in treating CTD-ILD is gradually being recognized, and the aforementioned studies provide positive real-world clinical data (23–25). Specifically, the combination of immunosuppressives with NTB has been shown to have a more significant advantage in protecting and improving lung function than NTB alone for treating PPF in patients with CTD-ILD. Furthermore, the combination does not result in a significant increase in the risk of adverse events. However, the current studies still have some limitations. Firstly, due to the small number of studies and sample sizes, it is not possible to accurately evaluate the efficacy and safety of RTX combined with NTB alone. Secondly, the predominance of SSc-ILD in the study population limits the discussion of combination therapy in ILD caused by different CTDs. Consequently, subsequent studies should expand the sample size and conduct subgroup analyses to enhance the reliability and generalizability of the conclusions.

## 5 Discussion

Rituximab, a monoclonal antibody targeting CD20 antigen, depletes B cells, thereby reducing the incidence of inflammatory responses and pulmonary fibrosis in CTD-ILD. It also maintains or improves lung function, enhances quality of life, and ultimately increases survival rates. The existing literature suggests that RTX is an effective treatment for CTD-ILD, however controversies persist. In typical circumstances, RTX can be employed as a salvage treatment following the failure of a first-line therapy comprising glucocorticoids and (or) immunosuppressants. However, some studies have demonstrated that RTX exhibits superior efficacy when used as an initial treatment compared to traditional regimens. Some studies have indicated that RTX has a superior safety profile to traditional immunosuppressive agents. However, because patients with refractory ILD have previously undergone treatment with glucocorticoids and (or) immunosuppressive agents, there is a potential for bias in assessing its safety profile. The adverse effects of RTX treatment are primarily infections, low serum IgG levels, neutropenia, and infusion reactions. However, some researchers have demonstrated that the administration of vaccines prior to treatment can reduce the incidence of adverse reactions. Recent recommendations from the European League Against Rheumatism (EULAR), the British Society for Rheumatology (BSR), and the American College of Rheumatology (ACR) on the management of CTD-ILD also state that RTX can be used as an initial or salvage regimen for CTD-ILD, particularly in patients with SSc-ILD (49–51). RTX can be used either when the disease progresses after first-line therapy or in the presence of RP-ILD, while considering the potential risk of infection. However, it should be noted that given the heterogeneity of CTD-ILD, the diverse clinical manifestations, the various subtypes of pathological organization, the HRCT model and serum autoantibodies, the efficacy of RTX treatment may be influenced. Additionally, the dosage and interval of RTX administration can impact treatment outcomes. However, the discrepancies observed in various studies may be attributed to several factors, including the limited sample size, the tendency for retrospective studies to be biased, and the potential for inter-observer variability in HRCT interpretation.

## 6 Conclusion

Rituximab has shown the efficacy and safety in treating CTD-ILD. However, controversy and limitations persist. Currently, research is primarily based on case reports and observational studies. Future studies should focus on conducting more long-term randomized controlled trials, clinical reviews, and meta-analyses to confirm the efficacy and safety of RTX treatment.
